# Prognostic value of cardiopulmonary exercise testing in patients with systemic sclerosis

**DOI:** 10.1186/s12890-019-1003-7

**Published:** 2019-11-29

**Authors:** Ralf Ewert, Till Ittermann, Dirk Habedank, Matthias Held, Tobias J. Lange, Michael Halank, Jörg Winkler, Sven Gläser, Horst Olschewski, Gabor Kovacs

**Affiliations:** 1 Department of Internal Medicine, University Greifswald, Greifswald, Germany; 2 Institute for Community Medicine, University Greifswald, Greifswald, Germany; 3 Department of Cardiology, DRK Kliniken Berlin, Berlin, Germany; 4 Missio Clinic, Würzburg, Germany; 5 Department of Internal Medicine II, University Medical Center Regensburg, Regensburg, Germany; 6 Department of Internal Medicine I, University Hospital of TU Dresden, Dresden, Germany; 7 PSL Pulmonary Study Center, Leipzig, Germany; 8 Department of Internal Medicine, Vivantes Kliniken Berlin, Spandau, Germany; 9 Department of Internal Medicine, Medical University of Graz, Division of Pulmonology, Graz, Austria; 10 Ludwig Boltzmann Institute for Lung Vascular Research, Graz, Austria

**Keywords:** Systemic sclerosis, Cardiopulmonary exercise, Pulmonary function, Prognosis, Pulmonary hypertension

## Abstract

**Background:**

Systemic sclerosis (SSc) is a severe rheumatic disease of the interstitial tissue, in which heart and lung involvement can lead to disease-specific mortality. Our study tests the hypothesis that in addition to established prognostic factors, cardiopulmonary exercise testing (CPET) parameters, particularly peak oxygen uptake (peakVO_2_) and ventilation/carbon dioxide (VE/VCO_2_)-slope, can predict survival in patients with SSc.

**Subjects and methods:**

We retrospectively assessed 210 patients (80.9% female) in 6 centres over 10 years with pulmonary testing and CPET. Survival was analysed with Cox regression analysis (adjusted for age and gender) by age, comorbidity (Charlson-Index), body weight, body-mass index, extensive interstitial lung disease, pulmonary artery pressure (measured by echocardiography and invasively), and haemodynamic, pulmonary and CPET parameters.

**Results:**

Five- and ten-year survival of SSc patients was 93.8 and 86.9%, respectively. There was no difference in survival between patients with diffuse (dcSSc) and limited cutaneous manifestation (lcSSc; *p* = 0.3). Pulmonary and CPET parameters were significantly impaired. Prognosis was worst for patients with pulmonary hypertension (*p* = 0.007), 6-min walking distance < 413 m (*p* = 0.003), peakVO_2_ < 15.6 mL∙kg^− 1^∙min^− 1^, and VE/VCO_2_-slope > 35. Age (hazard ratio HR = 1.23; 95% confidence interval CI: 1.14;1.41), VE/VCO_2_-slope (HR = 0.9; CI 0.82;0.98), diffusion capacity (Krogh factor, HR = 0.92; CI 0.86;0.98), forced vital capacity (FVC, HR = 0.91; CI 0.86;0.96), and peakVO_2_ (HR = 0.87; CI 0.81;0.94) were significantly linked to survival in multivariate analyses (Harrell’s C = 0.95).

**Summary:**

This is the first large study with SSc patients that demonstrates the prognostic value of peakVO_2_ < 15.6 mL∙kg^− 1^∙min^− 1^ (< 64.5% of predicted peakVO_2_) and VE/VCO_2_-slope > 35.

## Background

Systemic sclerosis (SSc) is a severe inflammatory disease of the interstitial tissue with clinical manifestations ranging from limited skin involvement to life-threatening effects on the heart, kidneys and lungs. SSc is a rare disease with an annual incidence in Europe of about 2 cases per 100,000 population, and a prevalence of about 10–25 per 100,000 [1, 2]. According to international registry studies [3], a high proportion of patients with SSc have interstitial lung disease (ILD), with or without pulmonary hypertension (PH), cardiac and gastrointestinal involvement. Cardiac, pulmonary and renal manifestations of SSc lead to an elevated disease-specific mortality [4–6]. Despite therapeutic progress, the mortality of patients with SSc is 3.5-fold higher than that of the general population – this factor has been stable over several decades [7].

Involvement of internal organs and joints typically results in impairment of exercise capacity, as measured by the 6-min-walk test (6-MWT) or cardiopulmonary exercise testing (CPET). In particular, CPET provides an important insight into exercise physiology, and has shown patients with SSc to have a lower cardiopulmonary exercise capacity, measured as peak oxygen uptake (peakVO_2_) [8] and as the relationship between ventilation and carbon dioxide output (VE/VCO_2_-slope) [9], compared with control individuals. Recent studies suggest that CPET can be used to determine whether the primary cause of exercise capacity limitation is cardiac or pulmonary in origin [10, 11]. Prognosis in SSc has not previously been assessed using CPET. However, studies in PH [12] and pulmonary arterial hypertension (PAH) [13] that included patients with SSc as a subgroup have suggested that CPET parameters may have prognostic value.

Against this background, we retrospectively assessed CPET parameters which could potentially predict survival. Analysis of a representative number of patients with SSc was made possible through the collaboration of multiple centres. Patients with SSc were subdivided into groups with and without interstitial pulmonary manifestations. We hypothesised that in addition to established prognostic factors – age, PH and ILD – CPET parameters, particularly peakVO_2_ and VE/VCO_2_-slope, can predict survival in patients with SSc.

## Methods

### Study design and participants

This study was a retrospective analysis of patients with SSc from a prevalent cohort. The patients were treated in four university hospitals (Greifswald, Regensburg, Dresden and Graz) and two expert centres (Missio Clinic Würzburg, and the Leipzig Pulmonary Study Center). All patients fulfilled the criteria of SSc or CREST syndrome (Calcinosis, Raynaud’s syndrome, Oesophageal dysmotility, Sclerodactyly, Telangiectasia; a subgroup of SSc with limited cutaneous manifestation [lcSSc]) according to current guidelines [14].

Patients without CPET data were excluded from the analysis, as were those with pulmonary diseases other than SSc (e.g. bronchial asthma, previous pulmonary surgery, or pulmonary emphysema visible in high-resolution computed tomography [HR-CT]). Patients with impaired systolic left ventricular function or relevant valvular disease other than tricuspid regurgitation (TR) were also excluded.

Patients with SSc were divided into two groups. Group 1 comprised patients with diffuse cutaneous SSc (dcSSc, *n* = 88). Group 2 (lcSSc, *n* = 122) included patients with lcSSc (including a subgroup presenting as CREST syndrome, *n* = 51). Pulmonary manifestation was assessed by HR-CT and pulmonary function testing as defined by the American College of Rheumatology/European League Against Rheumatism criteria [15]. Parenchyma involvement < 20% was considered to represent a limited manifestation. Extensive manifestation was defined as ≥20% parenchyma involvement. Patients with an uncertain extent of manifestation according to HR-CT were classified as extensive manifestation if forced vital capacity (FVC; as percentage of predicted [%predicted]) was < 70% of normal [16]. Co-morbidity was assessed using the Charlson index [17].

Follow-up and survival of all patients was documented from the first visit until June 30, 2016 (December 31, 2014 at Graz). Patients whose survival could not be documented at these dates were censored at the last day of contact. We defined three different follow-up times: 1) at time of diagnosis for the comparison between dcSSc and lcSSc (groups 1 and 2) and for demographic data such as age and gender; 2) at time of CPET for all other analyses except right heart catheterization (RHC) data; and 3) at time of RHC for analysis of the prognostic value of systolic right ventricular pressure (RV_sys_).

### Echocardiography

Resting echocardiography was performed by experienced physicians according to relevant guidelines [18, 19]. TR was classified according to American College of Cardiology/‌European Society of Cardiology (ESC) recommendations, and RV_sys_ was estimated by simplified Bernoulli equation via TR velocity (*v*) as RV_sys_ (mmHg) = 4*v*^2^, with the addition of 5 mmHg if the inferior vena cava was not dilated and there was visible respiratory variability, and 10 mmHg if the inferior vena cava was dilated or without respiratory variability.

### Pulmonary function and diffusion capacity

All centres assessed pulmonary function by spirometry, body plethysmography and measurement of diffusion capacity according to current standards [20–22]. Obstructive pulmonary disease was defined by forced expiratory volume in 1 second (FEV1)/FVC < 70%; restrictive pulmonary disease by total lung capacity (TLC) < 80%; and clinically relevant diffusion impairment by diffusion capacity of carbon monoxide (DLCO) < 60% of normal. Normal values for FEV1, FVC and TLC were calculated by the formulas published by our working group [23–25], and normal values for DLCO were taken from European Respiratory Society (ERS) formulas [26].

### Cardiopulmonary exercise testing

CPET was performed on a bicycle ergometer as a symptom-limited test. Performance and analysis methods have been described in detail previously [23, 27]. All centres started the test with a 3-min resting phase and unloaded cycling of 1–3 min (no unloaded phase was used at Graz), followed by a ramp protocol with 10–12.5 W∙min^− 1^ in two centres and a step-increment protocol with 12.5–16 W∙min^− 1^ in the other centres. All values were recorded as absolute values and percentage of normal, based on our reference values [23].

The 6-MWT was performed according to current American Thoracic Society guidelines [28].

### Right heart catheterisation

RHC was performed according to the guidelines of the ESC and the ERS [29] if clinical symptoms and echocardiographic criteria suggested possible PH. We applied the criteria defined in an expert consensus [30], which are based on clinical findings (progressive or unexplained dyspnoea, signs of right heart failure), echocardiography (RV_sys_ > 45 mmHg, right ventricular dilation) and DLCO (< 50%). All centres used the mid-thoracic level as the zero-pressure point. PH was defined according to ESC and ERS guidelines as mean pulmonary artery pressure (PAP_mean_) ≥25 mmHg, and PAH was defined as PAP_mean_ ≥ 25 mmHg, pulmonary artery wedge pressure (PAWP) ≤15 mmHg and pulmonary vascular resistance (PVR) > 3 Wood units (> 240 dyn∙s∙cm^− 5^) [31].

### Statistical analysis

Continuous variables, stratified by group status, are reported as median and interquartile range (IQR, in brackets). Categorical variables are reported as absolute numbers and percentages. Differences among groups were verified by Wilcoxon (continuous data) and χ^2^-tests (categorical data). Potential associations of group status and parameters from pulmonary function testing and CPET with mortality were tested using Cox regression models adjusted for age and gender. For group status the follow-up time was calculated based on the time of diagnosis; for the other variables the time of first examination defined the starting point.

Prediction models were determined using Cox regression models with age, gender, body mass index (BMI), and all parameters from pulmonary function testing and CPET as explanatory variables. For the final model, we eliminated variables by a backward selection procedure using a cut-off *p*-value of 0.1. The discrimination of these models was reported by Harrell’s C-statistic. Based on logistic regression models with the outcome “death: yes/no” we conducted receiver operating characteristic (ROC) analyses for selected variables. Kaplan–Meier curves were plotted for selected variables – for continuous variables, cut-off values were defined as the point which maximised the Youden index for the outcome “death”. The Youden index is defined as sensitivity + specificity − 1.

All analyses were carried out with Stata 14.1 (Stata Corporation, College Station, TX, USA).

### Ethical approval

The study was approved by the ethics committee of Greifswald University (No. 043/13a, study protocol and amendment of May 5th, 2015).

## Results

The study included 210 patients with SSc – demographic and clinical data are shown in Table 1. The majority of patients were women in both SSc groups, with group 2 (dcSSc) having a significantly lower proportion of women (73.9%) than group 1 (lcSSc, 86.1%; *p* = 0.03). The proportion of active smokers was < 20% in both SSc groups. There were no significant differences between SSc groups in co-morbidity status (Charlson index: 2 [IQR, 1–2] in both groups; *p* = 0.65) or in the proportion of patients with TR, assessed by echocardiography (80.3 vs 89.7%; *p* = 0.63). A significantly higher proportion of patients in group 1 had extensive ILD, compared with group 2 (27.1% vs 8.2%; *p* < 0.001).

**Table 1:** Demographic parameters

Parameter	N	Group 1 dcSSc	Group 2 lcSSc	p-value (group 1 vs. 2)
		n = 88	n = 122	
Age (years)	210	60 (51; 70)	62 (52; 70.2)	0.263
Female (n)	210	65 (73.9%)	105 (86.1%)	0.026
Time from first diagnosis to first visit at study centre (years)	203	4 (1; 7)	4 (1; 9)	0.471
Never-smoker (n)	137	57 (68.7%)	77 (70.6%)	
Ex-smoker (n)	25	14 (16.9%)	11 (10.1%)	
Smoker (n)	33	12 (14.5%)	21 (19.3%)	0.313
Charlson index	199	2 (1; 2)	1 (1; 2)	0.653
Height (cm)	210	165 (160; 175)	165 (160; 170)	0.943
Weight (kg)	210	71 (62; 85)	70 (62; 77)	0.206
BMI (kg∙m^−2^)	210	25.7 (23.0; 27.7)	24.8 (22.7; 28.4)	0.162
Limited ILD (n)	42	25 (29.4%)	17 (15.5%)	
Extensive ILD (n)	32	23 (27.1%)	9 (8.2%)	< 0.001

Data are presented as median (IQR) or n (%)

*BMI* Body mass index, *ILD* Interstitial lung disease, *IQR* Interquartile range, *dcSSc* disseminated cutaneous manifestation, *lcSSc* limited cutaneous manifestation

Pulmonary function parameters were significantly different between SSc groups, particularly with regard to FEV1%predicted (group 1, 90% [IQR, 77–104%]; group 2, 95% [IQR, 84–107%; *p* = 0.002]), and the proportion of patients with impaired FVC (< 70% of normal, 20.0% vs 8.6%; *p* = 0.02). There were no significant differences in diffusion parameters (DLCO %predicted and DLCO per alveolar volume [Krogh factor; KCO] %predicted; Table 2), or the proportion of patients with DLCO %predicted ≤60% (50.6% vs 37.8%; *p* = 0.08).

**Table 2:** Hemodynamic, pulmonary function and CPET parameters

Parameter	N	Group 1 dcSSc, n = 88	Group 2 lcSSc, n = 122	p-value (group 1 vs. 2)
Echocardiography available (n)	192	80 (90.9%)	112 (91.8%)	0.819
TR detected (n)	169	65 (80.3%)	104 (89.7%)	0.063
Estimated RV_sys_ (mmHg)	159	31 (25; 38)	32 (25; 45)	0.498
Right heart catheter available (n)	139	49 (55.7%)	90 (73.8%)	0.006
RAP_mean_ (mmHg)	135	5 (2; 7)	5 (3; 7)	0.653
PAP_mean_ (mmHg)	136	23 (16; 33)	21 (15; 30)	0.161
PAP_mean_ ≥ 25 mmHg	136	20 (42.6%)	32 (36.0%)	0.451
PAWP (mmHg)	136	9 (5; 13)	7 (6; 10)	0.059
PVR (Wood units)	134	2.68 (1.62; 5.34)	2.17 (1.49; 4.94)	0.587
Cardiac output (L∙min^−1^)	123	5.17 (4.40; 5.93)	4.94 (4.22; 5.88)	0.251
PAH (n)	134	13 (27.7%)	25 (28.7%)	0.895
TLC (% predicted)	205	93.8 (79.0; 107.0)	103.6 (90.9; 115.9)	0.026
VC (% predicted)	206	85.2 (75.8; 102.0)	100.5 (83.3; 109.0)	0.005
FVC (% predicted)	201	87 (75; 105)	97 (84; 110)	0.037
Proportion of patients with FVC ≤70% predicted	201	17 (20.0%)	10 (8.6%)	0.019
FEV1 (% predicted)	206	90 (77; 104)	95 (84; 107)	0.002
FEV1/FVC (%)	204	83 (78; 90)	79 (74; 86)	0.571
RV (% predicted)	204	104 (84; 124)	114 (95; 138)	0.468
RV/TLC (% predicted)	194	105,1 (92,6;122,2)	99,1 (87,1;111,7)	0.110
DLCO (% predicted)	190	60 (43; 77)	68 (45; 84)	0.616
Proportion of patients with DLCO ≤60% predicted	190	40 (50.6%)	42 (37.8%)	0.079
KCO (% predicted)	191	74.0 (56.6; 89.2)	71.8 (59.8; 86.3)	0.616
FVC (% pred.)/‌DLCO (% pred.)	185	1.48 (1.22; 1.94)	1.42 (1.22; 1.96)	0.719
6-MWD (m)	96	447 (372; 525)	423 (370; 478)	0.798
Maximum power (Watts)	209	84 (68; 100)	84 (68; 116)	0.723
Maximum power (% predicted)	209	87 (62; 117)	97 (75; 118)	0.180
VO_2_@AT in % of peakVO_2_ predicted	197	41.5 (31.8; 55.8)	41.0 (35.3; 47.1)	0.726
peakVO_2_ (mL∙min^−1^)	210	1171 (947; 1416)	1180 (899; 1476)	0.780
peakVO_2_ (% of predicted)	210	72.2 (58.4; 84.6)	75.2 (58.3; 90.0)	0.263
peakVO_2_/peakHR (L)	207	9.1 (7.2; 11.0)	9.0 (6.9; 10.8)	0.953
VE/VCO_2_-slope	200	31.6 (27.0; 40.0)	33.6 (28.0; 42.0)	0.117
VE/VCO_2_@rest	208	37 (32; 45)	38 (32; 43)	0.726
VE/VCO_2_@ AT	206	32 (29; 40)	34 (29; 42)	0.121
p_et_CO_2_@rest	205	30.8 (27.5; 34.0)	30.8 (26.5; 34.0)	0.953
p_et_CO_2_@AT	203	33.8 (29.8; 38.0)	33.0 (28.0; 37.9)	0.291
VE/MVV (%)	210	54.1 (43.5; 68.1)	55.2 (44.8; 63.4)	0.780
Proportion of VE/MVV > 80% (n)	210	9 (10.2%)	11 (9.0%)	0.768

Data are presented as median (IQR) or n (%)

*6-MWD* walking distance in 6 min, *CPET* Cardiopulmonary exercise testing, *DLCO* Diffusion capacity of carbon monoxide, *FEV1* Forced expiratory volume in 1 second, *FVC* Forced vital capacity, *IQR* Interquartile range, *KCO* Krogh factor (DLCO per alveolar volume), *lcSSc* limited cutaneous manifestation, *PAH* Pulmonary arterial hypertension, *PAP*_*mean*_ mean pulmonary arterial pressure (by right heart catheter); *RV*_*sys*_ Systolic pulmonary arterial pressure (by echocardiography), *PAWP* Pulmonary artery wedge pressure, *peakVO*_*2*_ peak oxygen uptake, *p*_*et*_*CO*_*2*_ End tidal pressure of carbon dioxide, *p*_*et*_*CO*_*2*_*@AT* End tidal pressure of carbon dioxide at anaerobic threshold, *PVR* Pulmonary vascular resistance, *RAP*_*mean*_ mean right atrial pressure, *RV* Residual volume, *TLC* Total lung capacity, *TR* Tricuspid regurgitation, *VC* Vital capacity, *VE/MVV* Ratio of ventilation to maximum voluntary ventilation, *VE/VCO*_*2*_*@AT* Ratio of ventilation to carbon dioxide output at anaerobic threshold, *VE/VCO*_*2*_*@rest* Ratio of ventilation to carbon dioxide output at rest, *VE/VCO*_*2*_*-slope* Slope of the relation between ventilation and carbon dioxide output, *VO*_*2*_*@AT* Oxygen uptake at anaerobic threshold, *VO*_*2*_*/HR* Ratio of oxygen uptake to heart rate

6-min-walking distance (6-MWD) was documented in 96 of 210 patients with SSc, with no significant difference between groups (*p* = 0.8). All CPET parameters tested were similar in the two SSc groups (e.g. peakVO_2_, 72.2% vs 75.2% of predicted; *p* = 0.3 and VE/VCO2-slope, 31.6 vs 33.6; *p* = 0.1). The overall correlation of 6-MWD and peakVO_2_ was weak (r = 0.2).

### Subgroup with right heart catheterisation

RHC data were available for 136 patients, of whom 52 had PH, including a subgroup of 38 patients with PAH. Patients with lcSSC more frequently underwent RHC (73.8% in group 1 vs 55.7% in group 2; *p* = 0.006). There were no significant differences between SSc groups in the proportion of patients with PH (42.6 vs 36.0; *p* = 0.45) or PAH (27.7% vs 28.7%; *p* = 0.9), or in haemodynamic parameters (Table 2). The subgroup with RHC had higher proportions of patients with extensive ILD and TR, higher mean estimated RV_sys_, and lower mean DLCO, FVC and 6-MWD. Most CPET parameters in the RHC group were worse compared with the non-RHC group (e.g. VE/VCO_2_-slope, 35 [IQR, 29–47] vs 29 (IQR, 26–33); peakVO_2_, 1087 (IQR, 824–1380) vs 1270 (IQR, 1097–1292) mL∙min^− 1^; both *p* < 0.001; see Additional file 1: Table S1).

### Subgroup with interstitial lung disease

All 195 patients with interpretable HR-CT were included in the subgroup analysis of pulmonary manifestation; of these, 191 patients had a complete pulmonary function test. The proportion of women was lower among patients with ILD (104 of 121; 86%) than among those without ILD (52 of 74; 74%, *p* < 0.01). Compared with patients without ILD, those with ILD had worse results in all pulmonary restriction and diffusion parameters, and more frequently underwent RHC. In addition, a higher proportion of patients with ILD had pulmonary limitation at exercise (defined as VE/MVV > 80%). There were no significant differences in co-morbidity or echocardiography, or in most haemodynamic and CPET parameters. A detailed comparison between patients with and without ILD is shown in Additional file 2: Table S2.

### Mortality

**Fig. 1:**
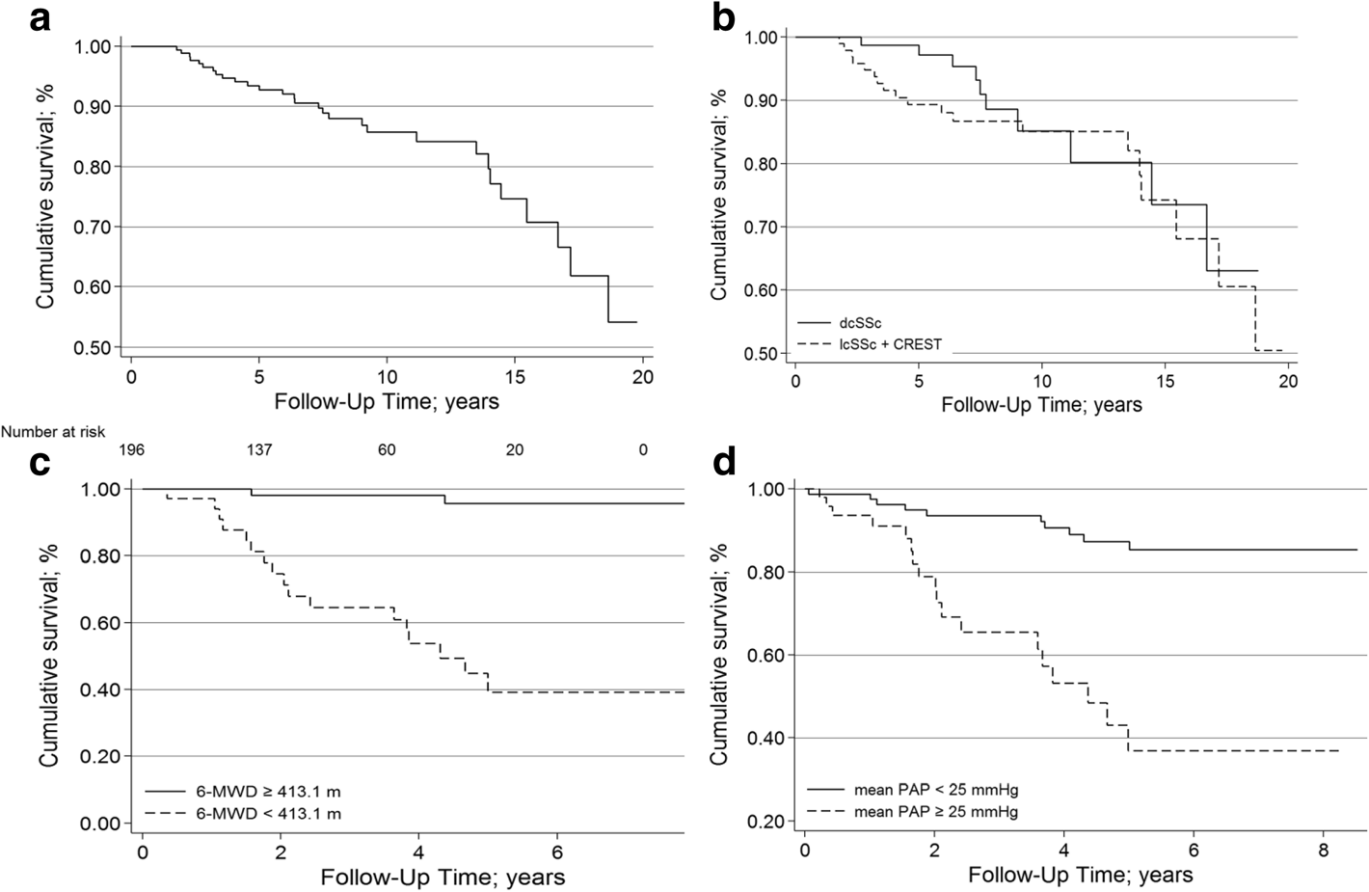
Survival of patients after first diagnosis of SSc (Kaplan–Meier analyses). **a** Overall. **b** According to limited or disseminated disease. Bold line: group 1 (dcSSc, n=88); dashed line: group 2 (n=122) comprising lcSSc (n=71) and CREST-syndrome (n=51). **c** Divided by 6-MWD, Youden index defining best cut-off at 413 m. **d** Divided by pulmonary hypertension. Bold line: PAPmean ≥25mmHg, dashed line: PAPmean <25mmHg. 6-MWD: 6 minute walking distance; CREST: Calcinosis, Raynaud´s syndrome, Oesophageal dysmotility, Sclerodactyly, Telangiectasia; dcSSc: disseminated cutaneous manifestation; lcSSc: limited cutaneous manifestation; PAPmean: mean pulmonary arterial pressure

The median follow-up after first diagnosis of SSc was 7.7 years, with a total of 1970 patient-years analysed. From first diagnosis, 5-year survival was 93.8%, and 10-year survival was 86.9% (Fig. 1a). There was no significant difference in survival between SSc groups (*p* = 0.3; Fig. 1b). In addition, there was no significant difference in survival between patients without ILD and those with extensive ILD (*p* = 0.1) or limited ILD (*p* = 0.25). In the subgroup of patients with RHC (*n* = 139), for whom analysis of PH was possible, a diagnosis of PH was associated with a significantly worse prognosis (*p* = 0.007, Fig. 1d).

### Prognostic factors

Cox regression analysis adjusted for age and gender determined that a number of factors were significantly associated with mortality (Table 3). Prognostic value was identified for age, Charlson index, body weight, BMI, extensive ILD, echocardiographic RV_sys_, and various haemodynamic parameters, pulmonary function and CPET. Moreover, 6-MWD was significantly associated with survival, with a walking distance of 413 m discriminating best (*p* = 0.003; Fig. 1c) between a favourable and a poor prognosis.

**Table 3:** Cox regression adjusted for age and gender

Parameter	Hazard ratio	95% confidence interval	*p*-value (bold: *p* < 0.05)
Demography			
age	1.07	1.02; 1.11	**0.002**
female	0.54	0.23; 1.27	
ex-smoker	2.80	0.77; 10.14	0.117
smoker	1.24	0.38; 4.04	0.724
Charlson index	1.41	1.16; 1.72	**0.001**
body height	0.98	0.93; 1.05	0.633
body weight	0.96	0.93; 0.99	**0.024**
BMI	0.88	0.79; 0.98	**0.019**
limited pulmonary manifestation	0.70	0.26; 1.94	0.497
extensive pulmonary manifestation	2.50	1.04; 6.00	**0.040**
Echocardiography			
RV_sys_	1.03	1.02; 1.05	**0.001**
Right heart catheterisation			
RAP_mean_	1.09	0.95; 1.24	0.200
PAP_mean_	1.04	1.01; 1.07	**0.002**
PAP_mean_ ≥ 25 mmHg	3.67	1.54; 8.75	**0.003**
PAWP	1.05	0.95; 1.15	0.347
PVR	1.22	1.11; 1.34	**0.001**
Cardiac output	0.45	0.28; 0.73	**0.001**
PAH	2.92	1.26; 6.75	**0.012**
Pulmonary function			
TLC	0.97	0.96; 0.99	**0.006**
VC (% pred.)	0.96	0.95; 0.98	**< 0.001**
FVC (% pred.)	0.97	0.96; 0.99	**0.001**
proportion of patients ≤70% predicted FVC	4.45	1.91; 10.35	**0.001**
FEV1 (% pred.)	0.98	0.96; 0.99	**0.005**
FEV1/FVC (%)	1.02	0.98; 1.06	0.241
RV (% pred.)	1.00	0.99; 1.01	0.451
RV/TLC (% pred.)	1.01	0.99; 1.04	0.189
DLCO (% pred.)	0.94	0.91; 0.96	**< 0.001**
proportion of patients ≤60% predicted DLCO	9.89	2.86; 34.19	**< 0.001**
KCO (% pred.)	0.95	0.93; 0.97	**< 0.001**
FVC (% pred.)/DLCO (% pred.)	2.25	1.49; 3.41	**< 0.001**
6-MWD	0.991	0.986; 0.997	**0.003**
CPET			
maximum power in Watts	0.95	0.94; 0.97	**0.001**
maximum power (% pred.)	0.96	0.95; 0.98	**< 0.001**
VO_2_@AT in % of peakVO_2_ predicted	0.99	0.97; 1.01	0.346
peakVO_2_	0.80	0.73; 0.88	**< 0.001**
peakVO_2_ (% pred.)	0.94	0.92; 0.96	**< 0.001**
VO_2_/HR	0.63	0.52; 0.75	**< 0.001**
VE/VCO_2_-slope	1.06	1.04; 1.09	**< 0.001**
VE/VCO_2_@rest	1.06	1.02; 1.10	**0.003**
VE/VCO_2_@AT	1.06	1.03; 1.09	**< 0.001**
p_et_CO_2_@rest	0.88	0.81; 0.95	**0.001**
p_et_CO_2_@AT	0.86	0.81; 0.92	**< 0.001**
VE/MVV (%)	1.01	0.98; 1.03	0.561
VE/MVV > 80%	1.43	0.42; 4.79	0.566

*6-MWD* Walking distance in 6 min, *BMI* Body mass index, *CPET* Cardiopulmonary exercise testing, *DLCO* Diffusion capacity of carbon monoxide, *FEV1* Forced expiratory volume in 1 second, *FVC* Forced vital capacity, *KCO* Krogh factor (DLCO per alveolar volume), *lcSSc* limited cutaneous manifestation, *PAH* Pulmonary arterial hypertension, *PAP*_*mean*_ Mean pulmonary arterial pressure (by right heart catheter), *PAWP* Pulmonary artery wedge pressure, *peakVO*_*2*_ peak oxygen uptake, *p*_*et*_*CO*_*2*_ End tidal pressure of carbon dioxide, *p*_*et*_*CO*_*2*_*@AT* End tidal pressure of carbon dioxide at anaerobic threshold, *pred.* predicted, *PVR* Pulmonary vascular resistance, *RAP*_*mean*_ mean right atrial pressure, *RV* Residual volume, *RV*_*sys*_ Right ventricular systolic pressure (by echocardiography), *TLC* Total lung capacity, *VC* Vital capacity, *VE/MVV* Ratio of ventilation to maximum voluntary ventilation, *VE/VCO*_*2*_*@AT* ratio of ventilation to carbon dioxide output at anaerobic threshold, *VE/VCO*_*2*_*@rest* ratio of ventilation to carbon dioxide output at rest, *VE/VCO*_*2*_*-slope* slope of the relation between ventilation and carbon dioxide output, *VO*_*2*_*@AT* oxygen uptake at anaerobic threshold, *VO*_*2*_*/HR* Ratio of oxygen uptake to heart rate

In a further step, the model was adjusted for BMI, age and gender and used to analyse all parameters of pulmonary function and CPET that had a significant association with survival (Table 4, model 1). In addition to age, in this model FVC, KCO and peakVO_2_ in mL∙kg^− 1^∙min^− 1^ were significantly linked to survival (Harrel’s C, 0.96). Exclusion of peakVO_2_ impaired the predictive value of the model (Harrel’s C, 0.84). In a calculation restricted to KCO, TLC and peakVO_2_, only peakVO_2_ remained associated with survival. A second model used peakVO_2_%predicted as a variable instead of peakVO_2_ in mL∙kg^− 1^∙min^− 1^: in this model, age, VE/VCO_2_-slope, KCO, FVC, and peakVO_2_%predicted had a significant association with survival (Table 4, model 2).

**Table 4:** Two different models for the calculation of predictive variables for survival

Mortality	Hazard ratio	*p*-value	95% Confidence interval	Harrell’s C	N
Model 1				0.96	148
Age	1.163	0.000	1070; 1264		
KCO	0.947	0.003	0.915; 0.981		
PeakVO_2_ (ml/kg/min)	0.653	0.000	0.529; 0.806		
FVC	0.942	0.000	0.913; 0.973		
Model 2				0.95	150
Age	1.272	0.000	1.143; 1.416		
VE/VCO_2_-slope	0.900	0.018	0.825; 0.982		
KCO	0.918	0.008	0.862; 0.978		
FVC	0.909	0.000	0.863; 0.957		
PeakVO_2_ (% pred.)	0.869	0.000	0.807; 0.937		

*FVC* Forced vital capacity, *KCO* Krogh factor (DLCO per alveolar volume), *peakVO*_*2*_ peak oxygen uptake, *pred.* predicted, *VE/VCO*_*2*_*-slope* slope of the relationship between ventilation and carbon dioxide output

**Fig. 2:**
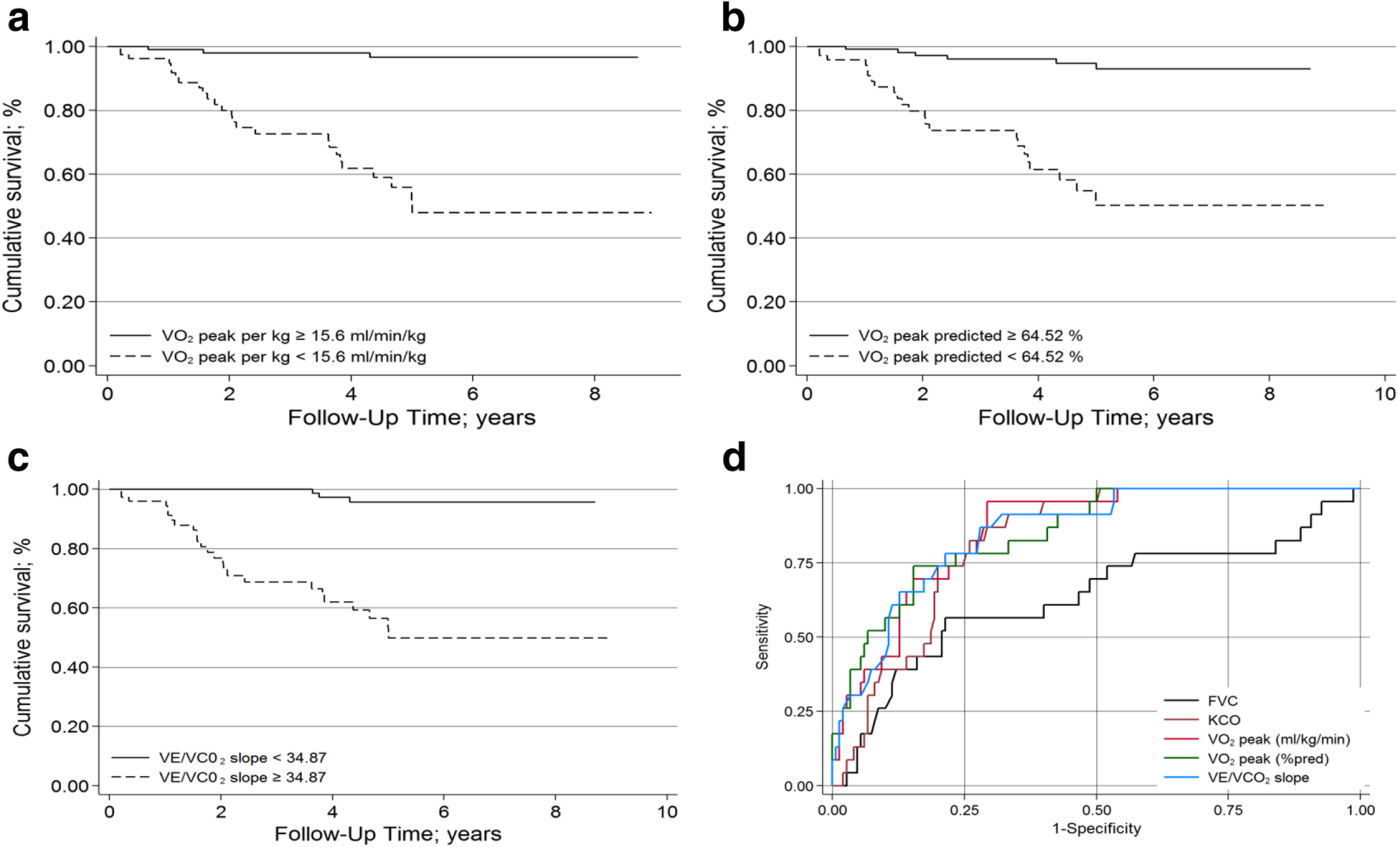
Survival and CPET parameters, Kaplan–Meier analysis (**a**-**c**), receiver operation characteristic. **d**. **a** peakVO2 in mL∙kg-1∙min-1. **b** peakVO2 as % of predicted normal value. **c** VE/VCO2-slope. **d** Receiver operation characteristic for selected parameters. FVC: forced vital capacity in % predicted (area under curve [AUC]=0.73; best cut-off [cut]=80%, Youden Index [Y]=0.30); KCO: Krogh factor (DLCO per alveolar volume in % predicted; AUC= 0.80, cut=62%, Y=0.54); peakVO2: peak oxygen uptake in mL∙kg-1∙min-1 (AUC=0.8, cut=15.6, Y=0.59); VE/VCO2-slope: slope of the relationship between ventilation and carbon dioxide output (AUC=0.8, cut=35, Y=0.57)

Finally, ROC analyses were conducted for the parameters peakVO_2_ and VE/VCO_2_-slope, and cut-off values were calculated (Fig. 2d). A peakVO_2_ of 15.6 mL∙kg^− 1^∙min^− 1^ (64.5% of predicted) and a VE/VCO_2_-slope of 34.9 had the highest discriminative value between favourable and poor prognoses (Fig. 2a-c).

## Discussion

The results of this study demonstrate for the first time in a large cohort of patients with SSc that CPET parameters (peakVO_2_, VE/VCO_2_-slope) and 6-MWD can predict survival.

Although there is some variation among previous studies (as detailed in Additional file 3: Table S3), these have in general found that peakVO_2_, oxygen uptake at the anaerobic threshold (VO_2_@AT) and the ratio of oxygen uptake to heart rate (VO_2_/HR) are lower in patients with SSc than reference or matched control values, while the ratio of ventilation to carbon dioxide output at the anaerobic threshold (VE/VCO_2_@AT) is higher [8, 9, 11, 32–37]. Our study confirmed these differences from reference values for pulmonary function, diffusion and CPET parameters.

The 5-year and 10-year survival rates from first diagnosis in our retrospective group of 210 patients with SSc were 93.8 and 86.9%, respectively. Overall, patients in group 1 (dcSSc) and group 2 (lcSSC) had similar 10-year survival rates (87% in both groups). This is consistent with results reported in the recent literature, with published 10-year survival rates of 93% in a Spanish study [4], 82% in a Canadian study [38], and 88% in an Italian study [39]. Earlier studies reported poorer 10-year survival rates, of 55% [40] and 54–67% [41].

In a Kaplan–Meier-analysis of our cohort according to pulmonary involvement, there was no significant difference for survival in patients with extensive or limited ILD compared with patients without ILD. However, Cox regression demonstrated a significantly higher risk of mortality in patients with extensive disease, compared with those without ILD (hazard ratio = 2.5; *p* = 0.04). This is in line with other published studies, which have shown significantly better survival rates in patients with moderate interstitial disease [16, 42] than in those with more extensive lung involvement, and with a meta-analysis that found the degree of interstitial changes to be an independent prognostic variable for mortality in SSc [43]. A recent study differentiated among subforms of ILD and showed that manifestation as usual interstitial pneumonia (UIP) has a 2.3-fold risk of mortality compared to manifestation as non-specific interstitial pneumonia (NSIP) [44]. Moreover, new drugs – rituximab [45, 46], mycophenolate [47], their combination [48], and nintedanib [49] – have the potential to provide an effective therapy for ILD. These therapies have been shown to improve parameters of pulmonary function that are related to prognosis, such as DLCO, DLCO/FVC and TLC [45, 50, 51], but to date no study has actually demonstrated improved survival in patients treated with immunosuppressive agents. Hence, there is a need for new parameters that better predict long time survival under immunosuppression [52].

Our analyses of subgroups as ILD/non-ILD and RHC/non-RHC found no relevant prognostic differences regarding CPET parameters. This might be caused by the heterogeneity of these groups or by a pre-selection bias. All study centres assessed CPET parameters as indication criteria for the performance of the RHC, and therefore nearly all CPET parameters were worse in the RHC group than in the non-RHC group (e.g. lower peakVO_2_ and higher VE/VCO_2_-slope). Similarly, the proportion of RHC in ILD was 84%, compared with 54% in non-ILD patients, preventing an evaluation of prognosis in these subgroups.

In accordance with the literature [53, 54] survival in our study was worse in patients with PH than among patients without PH. Multiple studies have shown that the prognosis of patients with ILD in addition to PH is even worse than in patients with PH alone (see Additional file 3: Table S3) [55–59]. It is notable that patients with PH who have SSc do not often suffer from PAH, but rather from PH due to left heart disease or PH due to lung disease (groups 1, 2 and 3 of the Nice classification, respectively) [31].

Our study has confirmed the prognostic significance of age, gender and pulmonary function parameters (vital capacity, TLC, FVC, FEV1, KCO, DLCO and quotient FVC/DLCO). Studies of these prognostic parameters, as well as meta-analyses describing patients with SSc with and without PH, have been reported previously [43, 60]. In particular, impaired DLCO and increased FVC/DLCO have a high sensitivity for predicting PH (particularly PAH) and have been included in several screening algorithms for PH in SSc [61–63].

In addition to these established parameters, our study showed a significant relationship between 6-MWD and survival in SSc. To our knowledge, this relationship has not previously been reported. The 6-MWD predicts prognosis in PAH [64], but has several limitations [65, 66]. In general, the use of 6-MWD in studies assessing pulmonary haemodynamics in patients with SSc has been recommended [67], but CPET is regarded as an alternative [68]. The weak correlation between 6-MWD and peakVO_2_ in our study may indicate that these two parameters identify different patients at risk. Previous 6-MWD studies have assessed subgroups of SSc. A recent meta-analysis of 6-MWD showed differences in walking distances between groups with or without PH or ILD [69]. For the subgroup of patients with SSc and ILD, the 6-MWD has been included in an algorithm for calculating mortality risk [70]. Similarly, in a meta-analysis of patients with SSc with PH, a shorter 6-MWD was associated with a worse prognosis [53], alongside age, gender, pericardial effusion, increased right atrial pressure, increased PAP_mean_, and reduced cardiac output. In contrast to our results, a retrospective study by Le Pavec et al. found no relationship between 6-MWD and survival in 70 patients with SSc with ILD and PH [71]. However, Zhao et al. found a 6-MWD of < 380 m to be an independent predictor of mortality in 190 patients with PH associated with various collagenoses [72]. This is consistent with our observations, and the difference from our cut-off value of < 430 m may result from our restricting the population to patients diagnosed with SSc, with or without PH.

The most important insight from our study may be the high prognostic relevance of CPET parameters for the survival of patients with SSc. The results confirm our hypothesis that peakVO_2_ and VE/VCO_2_-slope can predict survival. In addition, our study found this prognostic relationship in a cohort of patients of whom only a minority had PH or PAH. This is in contrast to previous studies, which have shown a prognostic relevance for CPET parameters only in patients with SSc who have PH or PAH [12, 13]. Multiple studies, including two analyses of patients with idiopathic PAH from our study group, have found peakVO_2_ and VE/VCO_2_-slope, among other parameters, to be related to survival [12, 13, 73, 74]. In a recent study of 226 patients with idiopathic PAH, peakVO_2_, VO_2_@AT, VO_2_/heart rate, p_et_CO_2_@rest, p_et_CO_2_@AT, VE/VCO_2_-slope and VE/VCO_2_@rest were related to survival in a univariate analysis (in a multivariate analysis only peakVO_2_ and VE/VCO_2_@rest were retained) [74]. Interestingly, CPET parameters can be sensitive in cases of pulmonary vasculopathy without manifested PH or PAH [10, 75, 76], because in these cases the integration of different cardiac, muscle and pulmonary pathologies in CPET parameters allows prognostication. Moreover, CPET can differentiate between predominantly cardiac and predominantly pulmonary manifestation, and increase the pre-test probability for PH [77]. In this way CPET may suggest specific therapeutic options.

### Limitations

Our retrospective study analysed a prevalent cohort of patients with SSc. The cohort was heterogeneous with respect to pulmonary pressure, ILD, and co-morbidities, which previous studies have found to affect the magnitude of changes in CPET parameters [32, 77, 78]. Although combined from six centres, the number of patients in our study was not high enough to separately analyse patients with PH and PAH. A slightly different CPET protocol was used in one centre, but this did not change the relevant CPET parameters [79]. However, despite substantial heterogeneity, we were able to identify highly significant prognosticators of survival which suggests robust results.

## Conclusions

Our study has demonstrated the prognostic value of the CPET parameters peakVO_2_ and VE/VCO_2_-slope in a large cohort of patients with SSc. Cut-off values of peakVO_2_ < 15.6 mL∙kg^− 1^∙min^− 1^ (< 64.5% of predicted) and VE/VCO_2_-slope > 35 predict worse survival. Further work is needed to determine whether the poor prognosis in these groups reflects the development of PH. If so, this would be of clinical importance, because while there is no specific SSc therapy, there are therapeutic options for the subgroup with PH. Therefore, peakVO_2_ or VE/VCO_2_-slope may increase the pre-test probability for PH, meaning that CPET results may suggest specific treatment.

## Supplementary information

**Additional file 1:** **Table S1** Demographic data in patients with and without right heart catheterization

**Additional file 2:** **Table S2** Demographic parameters in patients with and without ILD

**Additional file 3:** **Table S3** Cardiopulmonary exercise testing and prognostic parameters in SSc

## Data Availability

The database used to calculate the mortality and prognostic factors can be made available on request by Prof. Ralf Ewert via ralf.ewert@med.uni-greifswald.de. Availability of data from the participating six sites, however, might be limited by the patients’ consent and should be determined on a case-by-case basis.

## Abbreviations

∆VO_2_/HR: Difference between ratio of oxygen uptake / heart rate at rest and atmaximum exercise
6-MWD: 6-min-walking distance
6-MWT: 6-min-walk test
AHA: American Heart Association
AT: Anaerobic threshold
ATS: American Thoracic Society
BMI: Body mass index
CI: Confidence interval
CPET: Cardiopulmonary exercise testing
CREST: Calcinosis, Raynaud’s syndrome, Oesophageal dysmotility, Sclerodactyly, Telangiectasia
dcSSc: Disseminated cutaneous SSc
DLCO: Diffusion capacity of carbon monoxide
ERS: European Respiratory Society
ESC: European Society of Cardiology
FEV1: Forced expiratory volume in one second
FVC: Forced vital capacity
HR: Heart rate
HR-CT: High-resolution computed tomography
ILD: Interstitial lung disease
IQR: Interquartile range
KCO: Krogh factor (DLCO per alveolar volume)
lcSSc: SSc with limited cutaneous manifestation
LVEF: Left ventricular ejection fraction
NSIP: Non-specific interstitial pneumonia
PAH: Pulmonary arterial hypertension
PAP_mean_: Mean pulmonary arterial pressure (by right heart catheterization)
PAWP: Pulmonary artery wedge pressure
peakVO_2_: Peak oxygen uptake
p_et_CO_2_: End tidal pressure of carbon dioxide
p_et_CO_2_@AT: End tidal pressure of carbon dioxide at anaerobic threshold
PH: Pulmonary hypertension
PVR: Pulmonary vascular resistance
RAP_mea_: Mean right atrial pressure (by right heart catheterization)
RHC: Right heart catheterization
ROC: Receiver operating characteristic
RV: Residual volume
RV_sys_: Right ventricular systolic pressure (by echocardiography)
SD: Standard deviation
SSc: Systemic sclerosis
TLC: Total lung capacity
TR: Tricuspid regurgitation
UIP: Usual interstitial pneumonia
VE/MVV: Ratio of ventilation to maximum voluntary ventilation
VE/VCO_2_@AT: Ratio of ventilation to carbon dioxide output at anaerobic threshold
VE/VCO_2_@rest: Ratio of ventilation to carbon dioxide output at rest
VE/VCO_2_-slope: Slope of the relation between ventilation and carbon dioxide output
VO_2_/HR: Oxygen uptake per heart rate
VO_2_@AT: Oxygen uptake at anaerobic threshold
