# Supplementary material for: Prognostic value of cardiopulmonary exercise testing in patients with systemic sclerosis
Source: BMC Pulm Med. 2019 Nov 29;19:230. doi: 10.1186/s12890-019-1003-7 (PMC6884803; doi:10.1186/s12890-019-1003-7)
Supplement: Supplementary file 1 — Additional file 1: Table S1 Demographic data in patients with and without right heart catheterization [file 12890_2019_1003_MOESM1_ESM.docx]

Table S1 Demographic data in patients with and without right heart catheterization

| Parameter |  | **Group 1**  (no RHC) | **Group 2**  (with RHC) | **p-value**  **(group 1 vs. 2; bold: p<0.05)** |
| --- | --- | --- | --- | --- |
|  |  | N= 71 | N=139 |  |
| Age (years) | 195 | 57(47;68) | 64(54;71) | **0.010** |
| Female (n) | 210 | 55(77%) | 115(83%) | 0.358 |
| Never-smoker (n) | 121 | 49(72%) | 85(69%) |  |
| Ex-smoker (n) | 25 | 12(18%) | 13(10%) |  |
| Smoker (n) | 33 | 7(10%) | 26(21%) | 0.094 |
| Charlson index | 199 | 1(1;1) | 2(1;2) | **<0.001** |
| Height (cm) | 210 | 165(160;172) | 165(160;171) | 0.941 |
| Weight (kg) | 210 | 71(60;84) | 70(62;80) | 0.891 |
| BMI (kg∙m^‑^²) | 210 | 25.4(22.2;28.9) | 25.1(23;28.2) |  |
|  |  |  |  |  |
| ILD limited | 42 | 12(18%) | 35(28%) |  |
| ILD extensive | 32 | 7(10%) | 26(21%) | **<0.001** |
|  |  |  |  |  |
| Echocardiography available (n) | 192 | 58(82%) | 134(96%) | **<0.001** |
| TR detected (n) | 169 | 43 (74%) | 126(91%) | **0.002** |
| Estimated RV_sys_ (mmHg) | 159 | 30(25;31) | 33(26;54) | **0.009** |
|  |  |  |  |  |
| Right heart catheter available (n) |  |  |  |  |
| RAP_mean_ (mmHg) | 134 |  | 5(3;7) |  |
| PAP_mean_ (mmHg) | 135 |  | 21(15;33) |  |
| PAP_mean_ ≥25 mmHg | 52 |  | 52(38%) |  |
| PAWP (mmHg) | 135 |  | 8(5;11) |  |
| PVR (Wood units) | 133 |  | 2.3(1.5;5.0) |  |
| Cardiac output (L∙min^‑1^) | 122 |  | 5.01(4.3;5.88) |  |
|  |  |  |  |  |
| Pulmonary function |  |  |  |  |
| TLC (% predicted) | 205 | 102(91;113) | 96(84;112) | 0.180 |
| VC (% predicted) | 206 | 100(84;110) | 88(78;105) | **0.008** |
| FVC (% predicted) | 201 | 100(84;114) | 91(78;104) | **0.013** |
| Proportion of patients with FVC ≤70% predicted | 28 | 6(9%) | 21(16%) | 0.207 |
| FEV1 (% predicted) | 206 | 100(87;112) | 89(75;102) | **<0.001** |
| FEV1/FVC (%) | 204 | 84(79;91) | 80(75;85) | **0.001** |
| RV (% predicted) | 204 | 113(88;132) | 112(90;132) | 0.983 |
| RV/TLC (% predicted) | 194 | 104(95;119) | 100(88;1112) | 0.061 |
| DLCO (% predicted) | 190 | 70(60;85) | 57(39;78) | **<0.001** |
| Proportion of patients with DLCO ≤60% predicted | 82 | 18(26%) | 64(53%) | **<0.001** |
| KCO (% predicted) | 191 | 82(72;93) | 66(51;82) | **<0.001** |
| FVC (% pred.)/‌DLCO (% pred.) | 185 | 1.4(1.2;1.6) | 1.6(1.2;2.3) | **0.010** |
|  |  |  |  |  |
| 6-MWD (m) | 96 | 570(534;632) | 423(370;479) | **0.004** |
|  |  |  |  |  |
| Maximum power (Watts) | 209 | 100(84;125) | 75(68;100) | **<0.001** |
| Maximum power (% predicted) | 209 | 101(72;125) | 91(66;114) | **0.042** |
| VO_2_@AT in % of peakVO_2_ predicted | 197 | 48(38;56) | 27(19;41) | **<0.001** |
| peakVO_2_ (mL∙min^‑1^) | 210 | 1270(1097;1292) | 1087(824;1380) | **<0.001** |
| peakVO_2_ (% of predicted) | 210 | 79(66;90) | 71(53;85) | **0.001** |
| peakVO_2_/HR (L) | 200 | 10(8;11) | 8(7;10) | **<0.001** |
| VE/VCO_2_-slope | 200 | 29(26;33) | 35(29;47) | **<0.001** |
| VE/VCO_2_@rest | 208 | 36(32;41) | 38(32;44) | 0.127 |
| VE/VCO_2_@ AT | 206 | 30(28;33) | 37(30;44) | **<0.001** |
| p_et_CO_2_@rest | 205 | 31(28;33) | 31(26;35) | 0.687 |
| p_et_CO_2_@AT | 203 | 36(33;38) | 32(26;38) | **0.001** |
| VE/MVV (%) | 210 | 48(41;60) | 58(47;70) | **0.001** |
| Proportion of VE/MVV >80% (n) | 20 | 2(3%) | 18(13%) | **0.018** |

Data are presented as median (IQR) or n (%). *p* values <0.05 in bold

6-MWD: walking distance in 6 minutes; CPET: cardiopulmonary exercise testing; DLCO: diffusion capacity of carbon monoxide; FEV1: forced expiratory volume in one second; FVC: forced vital capacity; ILD: interstitial lung disease; IQR: interquartile range; KCO: Krogh factor (DLCO per alveolar volume); PAH: pulmonary arterial hypertension; PAP_mean_: mean pulmonary arterial pressure (by right heart catheter); RV_sys_: systolic pulmonary arterial pressure (by echocardiography); PAWP: pulmonary artery wedge pressure; peakVO_2_: peak oxygen uptake; p_et_CO_2_: end tidal pressure of carbon dioxide; p_et_CO_2_@AT: end tidal pressure of carbon dioxide at anaerobic threshold; PVR: pulmonary vascular resistance; RAP_mean_: mean right atrial pressure; RHC: right heart catheterization; RV: residual volume; TLC: total lung capacity; TR: tricuspid regurgitation; VC: vital capacity; VE/MVV: ratio of ventilation to maximum voluntary ventilation; VE/VCO_2_@AT: ratio of ventilation to carbon dioxide output at anaerobic threshold; VE/VCO_2_@rest: ratio of ventilation to carbon dioxide output at rest; VE/VCO_2_-slope: slope of the relation between ventilation and carbon dioxide output; VO_2_@AT: oxygen uptake at anaerobic threshold; VO_2_/HR: ratio of oxygen uptake to heart rate.
