# Supplementary material for: Prognostic value of cardiopulmonary exercise testing in patients with systemic sclerosis
Source: BMC Pulm Med. 2019 Nov 29;19:230. doi: 10.1186/s12890-019-1003-7 (PMC6884803; doi:10.1186/s12890-019-1003-7)
Supplement: Supplementary file 2 — Additional file 2: Table S2 Demographic parameters in patients with and without ILD [file 12890_2019_1003_MOESM2_ESM.docx]

Table S2 Demographic parameters in patients with and without ILD

| Parameter |  | **Group 1**  (no ILD) | **Group 2**  (ILD) | **p-value**  **(group 1 vs. 2; bold: p<0.05)** |
| --- | --- | --- | --- | --- |
|  | N | n=121 | n=74 |  |
| Age (years) | 195 | 60(49;70) | 62(54;71) | 0.255 |
| Female (n) | 195 | 104(86%) | 52(70%) | **0.008** |
| Never-smoker (n) | 121 | 82(75%) | 39(58%) |  |
| Ex-smoker (n) | 25 | 12(11%) | 13(19%) |  |
| Smoker (n) | 31 | 16(15%) | 15(22%) | 0.074 |
| Charlson index | 184 | 1(1;2) | 2(1;2) | 0.107 |
| Height (cm) | 185 | 165(160;170) | 167(160;174) | 0.317 |
| Weight (kg) | 185 | 69(61;78) | 70(62;80) | 0.554 |
| BMI (kg∙m^‑^²) | 185 | 24.7(22.3;28.8) | 25.2(23.1;27.4) | 0.945 |
|  |  |  |  |  |
| Echocardiography available (n) | 179 | 108(89%) | 71(96%) | 0.099 |
| TR detected (n) | 156 | 97(88%) | 59(82%) | 0.240 |
| Estimated RV_sys_ (mmHg) | 147 | 32(26;54) | 32(25;38) | 0.310 |
|  |  |  |  |  |
| Right heart catheter available (n) | 127 | 65 (54%) | 62 (84%) | **0.000** |
| RAP_mean_ (mmHg) | 123 | 5(3;7) | 5(2;7) | 0.573 |
| PAP_mean_ (mmHg) | 123 | 25(15;42) | 21(16;29) | 0.215 |
| PAP_mean_ ≥25 mmHg | 123 | 31(51%) | 20(32%) | **0.037** |
| PAWP (mmHg) | 123 | 8(6;10) | 8(5;11) | 0.571 |
| PVR (Wood units) | 122 | 2.62(1.53;7.28) | 2.29(1.58;4.01) | 0.340 |
| Cardiac output (L∙min^‑1^) | 111 | 4.86(4.15;5.88) | 5.05(4.5;5.84) | 0.407 |
|  |  |  |  |  |
| Pulmonary function |  |  |  | **0.000** |
| TLC (% predicted) | 190 | 106(93;114) | 91(75;104) | **0.000** |
| VC (% predicted) | 186 | 99(85;110) | 91(75;104) | **0.000** |
| FVC (% predicted) | 186 | 98(85;113) | 83(74;101) | **0.000** |
| Proportion of patients with FVC ≤70% predicted | 186 | 10(9%) | 16(21%) | **0.015** |
| FEV1 (% predicted) | 191 | 98(85;109) | 86(71;100) | **0.000** |
| FEV1/FVC (%) | 189 | 81(75;87) | 81(75;89) | 0.972 |
| RV (% predicted) | 189 | 117(97;138) | 101(79;121) | **0.001** |
| RV/TLC (% predicted) | 179 | 104(89;116) | 100(87;112) | 0.071 |
| DLCO (% predicted) | 177 | 67(52;84) | 51(36;70) | **0.000** |
| Proportion of patients with DLCO ≤60% predicted | 81 | 35(32%) | 46(67%) | **0.000** |
| KCO (% predicted) | 178 | 74(62;88) | 64(52;86) | **0.000** |
| FVC (% pred.)/‌DLCO (% pred.) | 172 | 1.4(1.2;1.9) | 1.6(1.3;2.3) | 0.071 |
|  |  |  |  |  |
| 6-MWD (m) | 86 | 436(352;508) | 420(371;468) | 0.381 |
|  |  |  |  |  |
| Maximum power (Watts) | 194 | 84(68;116) | 84(68;100) | 0.601 |
| Maximum power (% predicted) | 194 | 95(73;118) | 88(54;111) | **0.034** |
| VO_2_@AT in % of peakVO_2_ predicted | 181 | 38(24;49) | 32(21;43) | 0.124 |
| peakVO_2_ (mL∙min^‑1^) | 185 | 1172(918;1494) | 1150(920;1392) | 0.662 |
| peakVO_2_ (% of predicted) | 185 | 75(59;88) | 67(55;84) | 0.113 |
| peakVO_2_/HR (L) | 194 | 9(7;11) | 9(7;11) | 0.937 |
| VE/VCO_2_-slope | 186 | 32(29;41) | 33(29;42) | 0.319 |
| VE/VCO_2_@rest | 193 | 37(32;44) | 38(32;44) | 0.516 |
| VE/VCO_2_@ AT | 191 | 32(28;41) | 36(30;43) | 0.063 |
| p_et_CO_2_@rest (mmHg) | 190 | 30(27;33) | 31(28;35 | 0.138 |
| p_et_CO_2_@AT (mmHg) | 185 | 34(28;38) | 33(29;38) | 1.00 |
| VE/MVV (%) | 185 | 54(45;63) | 58(46;76) | 0.085 |
| Proportion of VE/MVV >80% (n) | 20 | 7(6%) | 13(18%) | **0.008** |

Data are presented as median (IQR) or n (%).

6-MWD: walking distance in 6 minutes; CPET: cardiopulmonary exercise testing; DLCO: diffusion capacity of carbon monoxide; FEV1: forced expiratory volume in one second; FVC: forced vital capacity; ILD: interstitial lung disease; IQR: interquartile range; KCO: Krogh factor (DLCO per alveolar volume); PAH: pulmonary arterial hypertension; PAP_mean_: mean pulmonary arterial pressure (by right heart catheter); RV_sys_: systolic pulmonary arterial pressure (by echocardiography); PAWP: pulmonary artery wedge pressure; peakVO_2_: peak oxygen uptake; p_et_CO_2_: end tidal pressure of carbon dioxide; p_et_CO_2_@AT: end tidal pressure of carbon dioxide at anaerobic threshold; PVR: pulmonary vascular resistance; RAP_mean_: mean right atrial pressure; RV: residual volume; TLC: total lung capacity; TR: tricuspid regurgitation; VC: vital capacity; VE/MVV: ratio of ventilation to maximum voluntary ventilation; VE/VCO_2_@AT: ratio of ventilation to carbon dioxide output at anaerobic threshold; VE/VCO_2_@rest: ratio of ventilation to carbon dioxide output at rest; VE/VCO_2_-slope: slope of the relation between ventilation and carbon dioxide output; VO_2_@AT: oxygen uptake at anaerobic threshold; VO_2_/HR: ratio of oxygen uptake to heart rate.
