# Supplementary material for: Prognostic value of cardiopulmonary exercise testing in patients with systemic sclerosis
Source: BMC Pulm Med. 2019 Nov 29;19:230. doi: 10.1186/s12890-019-1003-7 (PMC6884803; doi:10.1186/s12890-019-1003-7)
Supplement: Supplementary file 3 — Additional file 3: Table S3 Cardiopulmonary exercise testing and prognostic parameters in SSc [file 12890_2019_1003_MOESM3_ESM.docx]

**Supplementary Table 3 Cardiopulmonary exercise testing and prognostic parameters in SSc**

| **Reference** | **Author** | **Number of patients (N)** | **Main parameter** | **Result** |
| --- | --- | --- | --- | --- |
|  |  |  |  |  |
| **SSc and CPET** | | | | |
| 8 | Cuomo G 2010 | 46 | peakVO_2_ | Inversely correlates with heart and lung involvement |
| 9 | Rosato E 2014 | 40 | VE/VCO_2_-slope | Correlates with disease activity index |
| 10 | Boutou AK 2016 | 78 | peakVO_2_  VO2@AT  VE/VCO_2_-slope  p_ET_CO_2_  RV | Discriminates respiratory, left ventricular and pulmonary vascular limitation |
| 11 | Walkey AJ 2010 | 19 | AADO_2_ at exercise | Discriminates pulmonary-vascular and left ventricular limitation |
| 36 | Sudduth CD 1993 | 11 | peakVO_2_  VO_2_@AT  VO_2_/HR | Circulatory impairment |
| 49 | Michelfelder M 2017 | 54 | peakVO_2_  DLCO  FEV1%FVC | SSc and PAH patients, parameters do not discriminate coexisting interstitial lung disease |
| 58 | Vandecasteele E | 3185 (meta-analysis) | 6-MWD | PAH and ILD both shorten 6-MWD in SSc |
| 64 | Dumitrescu D 2010 | 30 | peakVO_2_  VO_2_@AT  VO_2_/HR  VE/VCO_2_@AT  p_ET_CO_2_@AT | VE/VCO_2_@AT and p_ET_CO_2_@AT distinguish pulmonary vasculopathy from left ventricular limitation |
| 65 | Reichenberger F 2009 | 33 | peakVO_2_ | Lower in exercise-induced PH |
| 66 | Dumitrescu D 2017 | 173 | peakVO_2_  VE/VCO_2_@AT | Both parameters highly correlated with PAP_mean_ |
| 67 | Morelli S 2000 | 18 | peakVO_2_  VO_2_@AT  VO_2_/HR | All associated with PH |
| 68 | Schwaiblmair M 1996 | 78 | peakVO_2_  DLCO  AADO_2_ at exercise | Discriminate lung involvement |
|  |  |  |  |  |
| **SSc patients vs normal subjects** | | | | |
| 32 | Chia EM 2016 | 25 | Δs' of right ventricle | Reduced in SSc vs normal |
| 33 | de Oliveira NC 2007 | 13 (female only) | peakVO_2_  DLCO | Reduced even without ILD |
| 34 | Hargardottir H 2010 | 11 | peakVO_2_  VE/VCO_2_-slope | Impaired in 9 of 11, inversely correlated with Interleukin-6 |
| 35 | Plazak W 2011 | 46 | peakVO_2_  VE/VCO_2_@AT  PAWP | PAWP>10 correlates with decreased peakVO_2_ and increased VE/VCO_2_-slope |
|  |  |  |  |  |
| **SSc and prognosis** | | | | |
| 37 | Swigris JJ 2009 | 83 | S_p_O_2_  S_a_O_2_ | Worse prognosis in SSc-ILD if saturation <89% or -4% at exercise |
| 42 | Poormoghim H 2011 | 91 | DLCO | In lcSSc later impaired than in dcSSc |
| 43 | Winstone TA 2014 | 1616 (meta-analysis) | DLCO  FVC | Predictive in both SSc and SSc-ILD |
| 44 | Lefevre G 2013 | 2244 (meta-analysis) | DLCO  6-MWD  PAP_mean_ | DLCO and pericardial effusion only prognostic factors in ILD-related PH |
| 45 | Trad S 2006 | 86 | DLCO  TLC  FVC | None of these parameters, but only PAH predicts survival in dcSSc |
| 47 | Launay D 2011 | 47 | DLCO | DLCO and pericardial effusion only prognostic factors in ILD-related PH |
| 52 | Coghlan JG 2014 | 73 | FVC/DLCO | DETECT and ASIG algorithms out-perform the ESC/ERS guidelines due to early inclusion of FVC/DLCO |
| 57 | Distler O, Behrens F | Expert consensus | 6-MWD  S_p_O_2_at exercise | recommended in prognosis assessment |
| 59 | Ryerson CJ 2015 | 156 | 6-MWD  FVC | Both predict 1-year mortality |
| 60 | Le Pavec J 2011 | 70 | 6-MWD | PH-Specific treatment does not change mortality in ILD-related PH |
| 61 | Zhao J 2017 | 190 | 6-MWD | Mortality in SSc-PAH higher than in Lupus erythematodes –PAH |
| 63 | Wensel R 2013 | 226 | peakVO_2_ | Predicts prognosis in PAH |

6-MWD: walking distance in 6 minutes; AADO_2_: alveolar-arterial difference of oxygen; CPET: cardiopulmonary exercise testing; DLCO: diffusion capacity of carbon monoxide;FEV1: forced expiratory volume in one second; FVC: forced vital capacity; ILD: interstitial lung disease; KCO: Krogh factor (DLCO per alveolar volume); lcSSc: limited cutaneous manifestation; PAH: pulmonary arterial hypertension; PAP_mean_: mean pulmonary arterial pressure; PAWP: pulmonary artery wedge pressure; peakVO_2_: peak oxygen uptake; p_et_CO_2_: end tidal pressure of carbon dioxide; p_et_CO_2_@AT: end tidal pressure of carbon dioxide at anaerobic threshold; VE/VCO_2_@AT: ratio of ventilation to carbon dioxide output at anaerobic threshold; VE/VCO_2_-slope: slope of the relation between ventilation and carbon dioxide output; VO_2_@AT: oxygen uptake at anaerobic threshold; VO_2_/HR: ratio of oxygen uptake to heart rate.
